# Overexpression of *VrUBC1*, a Mung Bean E2 Ubiquitin-Conjugating Enzyme, Enhances Osmotic Stress Tolerance in *Arabidopsis*

**DOI:** 10.1371/journal.pone.0066056

**Published:** 2013-06-18

**Authors:** Eunsook Chung, Chang-Woo Cho, Hyun-Ah So, Jee-Sook Kang, Young Soo Chung, Jai-Heon Lee

**Affiliations:** Department of Genetic Engineering, College of Natural Resources and Life Science, Dong-A University, Busan, Republic of Korea; National Taiwan University, Taiwan

## Abstract

The ubiquitin conjugating enzyme E2 (UBC E2) mediates selective ubiquitination, acting with E1 and E3 enzymes to designate specific proteins for subsequent degradation. In the present study, we characterized the function of the mung bean *VrUBC1* gene (*Vigna radiata UBC 1*). RNA gel-blot analysis showed that *VrUBC1* mRNA expression was induced by either dehydration, high salinity or by the exogenous abscisic acid (ABA), but not by low temperature or wounding. Biochemical studies of VrUBC1 recombinant protein and complementation of yeast *ubc4/5* by *VrUBC1* revealed that *VrUBC1* encodes a functional UBC E2. To understand the function of this gene in development and plant responses to osmotic stresses, we overexpressed *VrUBC1* in Arabidopsis (*Arabidopsis thaliana*). The *VrUBC1*-overexpressing plants displayed highly sensitive responses to ABA and osmotic stress during germination, enhanced ABA- or salt-induced stomatal closing, and increased drought stress tolerance. The expression levels of a number of key ABA signaling genes were increased in *VrUBC1*-overexpressing plants compared to the wild-type plants. Yeast two-hybrid and bimolecular fluorescence complementation demonstrated that VrUBC1 interacts with AtVBP1 (*A. thaliana* VrUBC1 Binding Partner 1), a C3HC4-type RING E3 ligase. Overall, these results demonstrate that *VrUBC1* plays a positive role in osmotic stress tolerance through transcriptional regulation of ABA-related genes and possibly through interaction with a novel RING E3 ligase.

## Introduction

Plants are frequently exposed to stressful environmental conditions that can significantly impact plant growth and development. Drought and salinity stresses are two of the most important environmental stresses, and are responsible for dramatic reductions in crop yield worldwide [1]. To tolerate such unfavorable conditions, plants have evolved a variety of strategies such as reduced transpiration, osmolyte accumulation and removal of toxic molecules including denatured proteins and reactive oxygen species [2], [3].

The ubiquitin/proteasome system is the main pathway for selective protein degradation in eukaryotic cells [4]. Ubiquitination has important functions in many aspects of plant growth and development, including phytohormone and light signaling, embryogenesis, organogenesis, leaf senescence, and plant defense [5]–[8]. Ubiquitin-dependent protein degradation consists of two discrete steps. First, the target protein is tagged by the attachment of multiple ubiquitin molecules for recognition by the 26S proteasome complex. Second, the tagged protein is degraded by the 26S proteasome, releasing free and reusable ubiquitin molecules. The first step of ubiquitination involves three stages: the activation of ubiquitin catalyzed by the ubiquitin-activating enzyme E1, the transfer of ubiquitin to a ubiquitin-conjugating enzyme (UBC) E2, and the ligation of ubiquitin to the protein substrate by the direct transfer of ubiquitin from E2 or from a protein ligase E3 [9], [10]. In the *Arabidopsis thaliana* genome, there are 2 E1s, 37 E2s and more than 1,300 genes predicted to encode E3s [11], [12]. Thus, E3 and E2 are considered to play a crucial role in the specificity of ubiquitination.

The E2s were originally defined as proteins capable of accepting ubiquitin from an E1 through a thioester linkage via a cysteinyl-sulfhydryl group [13]. The E2s exist as a multigene family; there are 11 E2s in the *Saccharomyces cerevisiae* genome, and 50 E2s in the human genome. All E2s contain a conserved domain of about 16 kDa called the UBC domain, which is a ∼150-amino-acid catalytic core [14]. The UBC domain also interacts with the E3 enzyme and with the substrate [15]. *In vitro* UBC E2 activity has been demonstrated for wheat TaUBC7 [16], and for 17 Arabidopsis UBC E2s [12], [16]–[18]. The C-terminal region of UBC E2 determines the E2 substrate specificity and thus provides selectivity to the ubiquitin system [19]. Expression of *UBC E2* genes from a number of plant species are regulated by tissue and/or development [8] and also by environmental conditions [20]–[24]. Overexpression of soybean *GmUBC2* and peanut *AhUBC2* resulted in improved drought tolerance in Arabidopsis [21], [22]. In the *UBC E2* overexpressing plants, expression of stress-responsive genes was upregulated and proline levels were increased, compared to the wild-type [21], [22]. Arabidopsis *UBC32* was shown to be involved in salt stress response through brassinosteroid-mediated siganlling [23]. However, their precise molecular functions in abiotic stress signaling responses have not been clearly identified.

Although many E2 functions remain unknown, E3 ligases have been extensively studied in development and in signaling responses during abiotic stress [25]. E3 ligases may act as either negative or positive regulators in stress signaling. Hot pepper (*Capsicum anuum) CaPUB1* and Arabidopsis *PUB22/PUB23*, which encode U-box E3 ligases, act as negative regulators in osmotic stress tolerance [26], [27]. Arabidopsis *HOS1*, encoding a variant RING finger E3 ubiquitin ligase, negatively regulates transcription factor *ICE1* and modulates downstream cold-responsive gene transcription [28], [29]. ABI3-interacting protein (AIP2), an E3 ligase, plays a negative role in osmotic stress responses by targeting ABI3 for degradation [30]. Arabidopsis DRIP1/DRIP2, C3HC4-type RING E3 ligases, target DREB2A for ubiquitination and thus act as negative regulators in the response to drought stress [31]. It has been shown that the novel RING E3 ligase KEG probably targets ABI5 for degradation [32]. Also, *AtCHIP* may function upstream of *PP2A* in stress-responsive signal transduction pathways under conditions of low temperature or darkness [33]. By contrast, E3 ligases such as *XERICO* [34], *RHA2a* [35], *SDIR1* [36] and *AtAIRP1/AtAIRP2* [37], [38] act as positive regulators of ABA signaling [34], [35], [36]. Collectively, these results suggest a linkage between protein ubiquitination and stress responses in plants.

In this study, *VrUBC1* encoding UBC E2 was cloned from mung bean and its expression was responsive to dehydration, salinity, and ABA treatment. Transgenic experiments indicated that overexpression of *VrUBC1* resulted in osmotic stress tolerance-associated phenotypes, such as increased sensitivity to ABA and enhanced tolerance to dorught stress by inducing stomatal closure in plants. Furthermore, we found that a potential target of VrUBC1 in the *35S:VrUBC1* Arabidopsis is AtVBP1, a novel C3HC4-type E3 ligase, which might be involved in the ABA-mediated signaling cascade. Collectively, the results presented in this report suggest that the VrUBC1 plays a positive regulator in osmotic stress tolerance possibly interacting with AtVBP1 in ABA-mediated osmotic stress responses in Arabidopsis.

## Results

### Molecular Characterization of *VrUBC1*, Encoding an UBC E2, Induced by Abiotic Stress

We previously isolated and identified a number of mung bean cDNA clones differentially expressed under abiotic stress conditions [39]. One of the clones, *MLT113*, showed a high level of DNA sequence identity to the *UBC E2* gene designated as *VrUBC1* [39] (Genbank accession number, FJ436357). The full-length cDNA of *VrUBC1* is 825 bp long with a 447-bp open reading frame (ORF) encoding a 148 amino acid polypeptide (predicted molecular weight; 16.5 kDa.). UBC E2s are classified into 4 groups: class I with only the catalytic domain containing a Cys (cysteine) residue for the formation of the thioester bond with ubiquitin; class II with the C-terminal extension; class III with the N-terminal extension; and class IV with both the N- and C-terminal extensions [9]. The deduced amino acid sequence of *VrUBC1* consists of the catalytic domain of a class I type UBC E2 (Figure S1A in File S1). Multiple sequence alignment of the deduced amino acids of *VrUBC1* shows that VrUBC1 protein shares high sequence similarity with Arabidopsis AtUBC10 (97% identity) and other eukaryote UBCs such as the yeast ScUBC5 (78%), and human HsUBCH5D (81%) (Figure S1A in File S1). To compare VrUBC1 to 25 UBC E2s from the diverse organisms and all the Arabidopsis UBC E2s, the phylogenetic tree was constructed (Figure S1B in File S1). VrUBC1 protein shows a high degree of sequence similarity to the Arabidopsis E2 subgroup IV clade members out of 16 clades, such as AtUBC10 (At5g53300, 97%), AtUBC9 (At4g27960, 96%), AtUBC8 (At5g41700, 96%), AtUBC11 (At3g08690, 95%), AtUBC28 (At1g64230, 90%), AtUBC30 (At5g56150, 89%), AtUBC29 (At2g16740, 86%) and AtUBC12 (At3g08700, 79%) (Figure S1B in File S1) [12]. This indicates that VrUBC1 can be grouped into the largest Arabidopsis E2 subgroup IV clade. Phylogenetic analysis revealed that VrUBC1 is more similar to the E2s of higher plants (90–100% identity), lower plants (82–85%), fungi (78–80%) and animals (79–81%) than to other Arabidopsis UBCs (Figure S1B in File S1). This implies that class I type UBC E2s are highly conserved and may have similar functions in diverse organisms.

**Figure 1:**
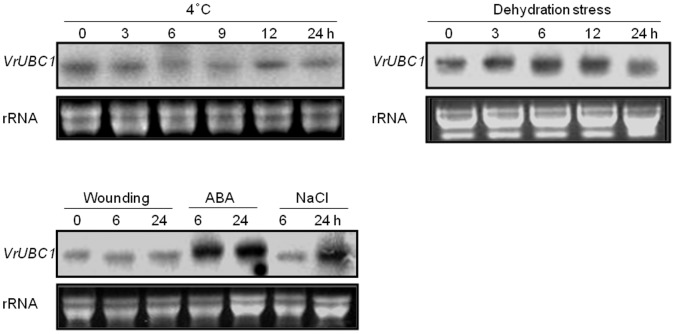
Northern blotting of *VrUBC1* in mung bean. *VrUBC1* RNA expression in mung bean leaves exposed to low temperature (4°C), dehydration, wounding, ABA (100 µM), or salt stress (100 mM NaCl). Twenty micrograms of total RNA was loaded in each lane**.** Following electrophoresis, RNA was transferred to a nylon membrane and hybridized with a probe specific for *VrUBC1*. Equal loading of the total RNA (20 µg) was confirmed by EtBr staining, shown as rRNA below the signal panel.

We next used Northern blotting to test *VrUBC1* expression in response to abiotic stress in mung bean plants (Figure 1). RNA expression level of *VrUBC1* was not shown to be changed during low temperature stress (Figure 1). *VrUBC1* RNA expression was monitored during dehydration, wounding, salinity and ABA treatment (Figure 1). Transcript level of *VrUBC1* gradually was shown to increase by dehydration stress (Figure 1). Reduced expression after 24 h dehydration stress may be resulted from the degradation of total RNAs due to the severe damage on the mung bean (Figure 1). Wounding stress did not affect its expression, but its expression was strongly induced at the 6 h and 24 h time points following application of ABA (100 µM) (Figure 1). Salinity (100 mM NaCl) stress also induced *VrUBC1* RNA expression at 24 h (Figure 1). Specific regulation of *VrUBC1* by the osmotic stress signals indicates that *VrUBC1* may be involved in molecular responses to dehydration, and high salinity stress through ABA pathway in plant.

### Complementation of *ScUBC4/5* by *VrUBC1* and *in vitro* Ubiquitin Conjugation of the VrUBC1 Protein

**Figure 2:**
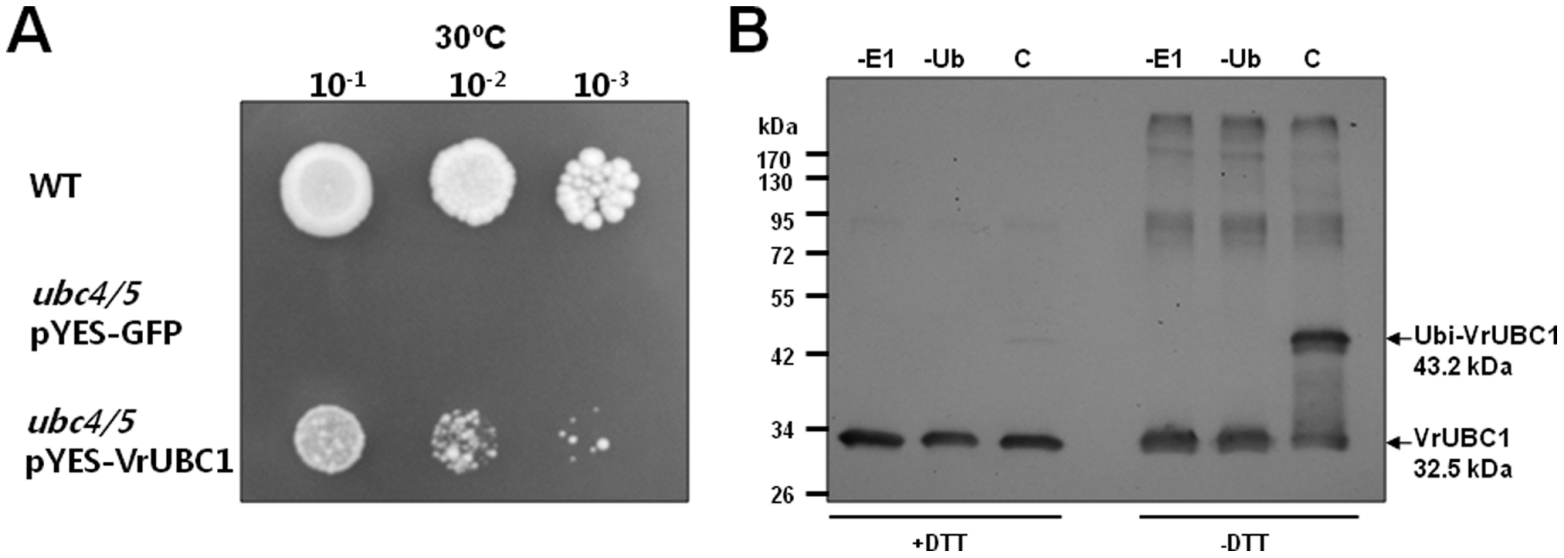
Yeast complementation and thioester formation of VrUBC1. (A) For the *ubc4/5* complementation test, the yeast *ubc4/5* double mutant was transformed with pYES-GFP or pYES-VrUBC1, and selected on uracil-lacking SD medium plates (SD/−Ura). The wild-type (WT) and the *ubc4/5* transformants harboring pYES-GFP or pYES-VrUBC1 were grown to an OD600 of 1.0, and 10 µl aliquots of different dilutions (10^−1^, 10^−2^, 10^−3^) were spotted onto SD/−Ura plates and grown for 3 d at 30°C. (B) Thioester formation of VrUBC1. VrUBC1 forms DTT-sensitive ubiquitin adducts. *In vitro* ubiquitination reactions after 5-min at 37°C were treated with DTT or 4 M urea (-DTT). Reactions were resolved by SDS-PAGE and western blots were performed with with anti-His_6_ antibodies.

Yeast ScUBC4 and ScUBC5 are typical class I E2s [9]. Class I E2s from the nematode, fruit fly, fungus, and cotton can complement *ScUBC4/5* function in yeast [40]–[43]. For example, the *Caenorhabditis elegans UBC2* and the cotton *GhUBC1/2* have been shown to complement *ScUBC4* and *ScUBC5* function in the double *ubc4/5* mutants [41], [43]. Given the strong amino acid conservation between VrUBC1 and the yeast ScUBC4/5, it is tempting to speculate that these proteins are functionally analogous (Figure S1 inFile S1). To examine this, we conducted a yeast complementation experiment to determine whether *VrUBC1* can complement *ScUBC4* and *ScUBC5* function in yeast (Figure 2A). Double mutants lacking *ScUBC4* and *ScUBC5* were used for the complementation assay because the *ubc4* or *ubc5* single mutants do not show any growth defect [44]. The transformed *ubc4/5* mutant harboring pYES-VrUBC1 grew much faster than the mutant transformed with the pYES-GFP vector (Figure 2A). However, the mutants with pYES-VrUBC1 grew slower than the wild-type yeast strains (Figure 2A). Thus, *VrUBC1* can partially complement the function of the yeast *ScUBC4* and *ScUBC5* genes.

The region surrounding this Cys has been confirmed as the E2 active site motif [HPN(I/V)(X)_3-4_GX(I/V/L)C(I/L)X(I/V)(I/L)], which is found in the majority of the identified E2s (Figure S1A in File S1) [45]. The active Cys site at position 85 of VrUBC1 is predicted to form a thioester bond with the C-terminal Gly residue of ubiquitin (Figure S1A in File S1). To examine the ubiquitin-conjugation activity of the VrUBC1 protein *in vitro*, we produced recombinant VrUBC1 protein in *Escherichia coli*. VrUBC1 (16.5 kDa) fused to the C-terminus of thioredoxin (Trx; 13 kDa) was expressed with a C-terminal His-tag (3 kDa). To determine the factors involved in the formation of the thioester bond between ubiquitin (10.7 kDa) and VrUBC1 (32.5 kDa), the reactions were incubated with or without E1, or ubiquitin and subjected to western blotting with anti-His_6_ antibodies to detect Ubi-VrUBC1 (43.2 kDa) in the presence or absence of DTT (Figure 2B). E1 and ATP were essential for the formation of the thioester bond between ubiquitin and VrUBC1 (Figure 2B). Immunoblots with anti-His_6_ antibodies show the presence of a DTT-sensitive ubiquitin adduct for VrUBC1 since the adducts were lost in the presence of a thiol-reducing agent (DTT) (Figure 2B). This indicates that a thioester linkage was formed between ubiquitin and E2. Taking the results together, we conclude that the thioester bond between ubiquitin and VrUBC1 is formed *in vitro*. Taken together, it can be inferred that *VrUBC1* encodes a functional E2 from the deduced sequence analogy, yeast complementation and *in vitro* ubiquitination assays.

### Transgenic Arabidopsis Plants Overexpressing *VrUBC1* Show Retarded Germination and Improved Tolerance to Osmotic Stress

**Figure 3:**
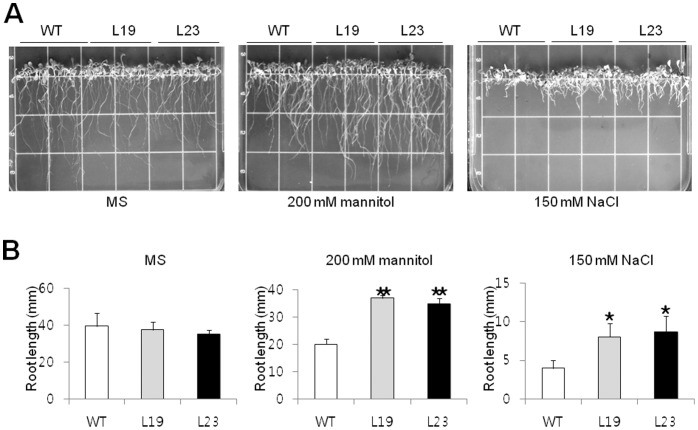
Tolerance tests of wild-type and *35S:VrUBC1* Arabidopsis transgenic plants under osmotic stress or ABA conditions. (A) Ten-d-old seedlings of the wild-type and *35S:VrUBC1* Arabidopsis transgenic lines (L19 and L23) were transferred to MS medium containing 2% (w/v) sucrose and 0.8% (w/v) phytoagar supplemented either mannitol (200 mM) or with NaCl (150 mM). (B) Root length was monitored after 10 days. The values are the means ± SD (n = 3). This experiment was carried out three times with consistent results.

To investigate the function of *VrUBC1* in abiotic stress responses, we overexpressed *VrUBC1* in Arabidopsis. Independent transgenic lines showed the constitutive expression of *VrUBC1* based on RT-PCR (Figure S2A in File S1). We chose two independent T3 homozygous lines (L19 and L23) showing moderate RNA expression levels of *VrUBC1* based on the quantitative real-time qRT-PCR analysis (Figure S2B in File S1). The overexpressing transgenic plants (L9) with the highest *VrUBC1* expression did not show significant much difference in phenotype compared to the wild-type plants under nonstress conditions based on the statistical analyses (Figure S2C, S2D in File S1). To determine whether *VrUBC1* overexpression affected osmotic stress tolerance in Arabidopsis, growth rates of the wild-type and *35S:VrUBC1* transgenic seedlings were compared under osmotic stress conditions (Figure 3). Ten-d-old seedlings of the wild-type and transgenic plants overexpressing *VrUBC1* were transferred to media containing 150 mM NaCl or 200 mM mannitol and incubated for 10 d (Figure 3). Difference in root length of *VrUBC1* overexpressing plants and the wild-type plants is not statistically significant under nonstress conditions (Figure 3A). Under osmotic stress conditions, the *35S:VrUBC1* overexpressing plants displayed better shoot and root growth in mannitol and NaCl stress conditions compared to the wild-type plants (Figure 3A). *VrUBC1* overexpression lines showed significantly increased root length compared to that of the wild-type under the stress conditions (Figure 3B). This result implies that the *VrUBC1* gene acts a positive regulator of osmotic stress tolerance.

**Figure 4:**
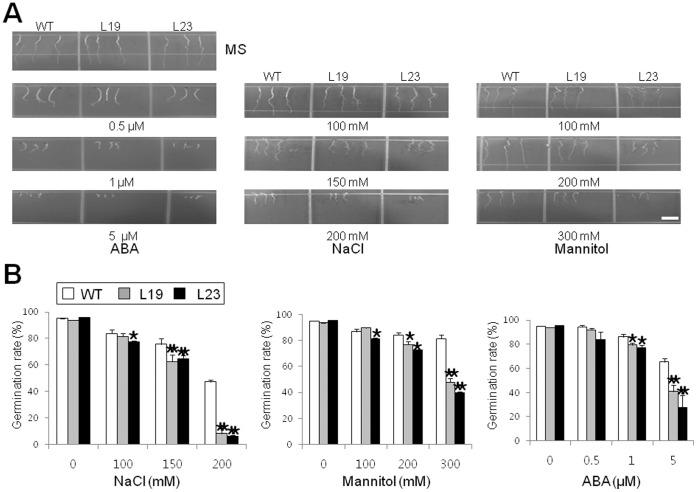
Germination rates of the wild-type and *35S:VrUBC1* Arabidopsis transgenic plants under osmotic stress or ABA conditions. (A) Photographs of seedlings at 4 d after the end of stratification. Seeds were germinated on MS medium containing different concentrations of ABA (0, 0.5, 1, or 5 µM), NaCl (0, 100, 150 or 200 mM), or mannitol (0, 100, 200, or 300 mM) and were incubated at 4°C for the stratification (3 d). (B) Seed germination percentage of the indicated lines grown on different concentration of NaCl, mannitol, or ABA, was recorded at 4 d after the end of stratification. Data show the mean ± SD of three replicates. At least 100 seeds per line were measured in each replicate. Asterisks indicate the significance of the difference from the values between the wild-type and the *35S:VrUBC1* Arabidopsis transgenic plants as determined by Student’s *t* test (*0.01 ≤ P<0.05, **P<0.01).

To further examine the role of *VrUBC1* in relation to ABA and osmotic stress responses during germination, we carried out ABA, NaCl, and mannitol dose-response germination analyses of the wild-type and *35S:VrUBC1* plants (Figure 4A). We observed a small reduction in the germination rate of *35S:VrUBC1* plants compared to the wild-type at low concentrations of ABA (0.5 µM), NaCl (100 mM), and mannitol (100 mM) and a more substantial reduction at higher concentrations (Figure 4B). Collectively, these results indicate that ectopic expression of *VrUBC1* represses seed germination and increases seedling tolerance of osmotic stress and enhance their response to ABA. Stress-inducible and developmental specific promoters instead of the constitutive promoter may be more applied to maximize the enhancement of stress tolerance in transgenic plants.

### Enhanced Tolerance and ABA-signalling Upstream Gene Expression under the Drought Stress Condition in *35S:VrUBC1* Transgenic Plants

**Figure 5:**
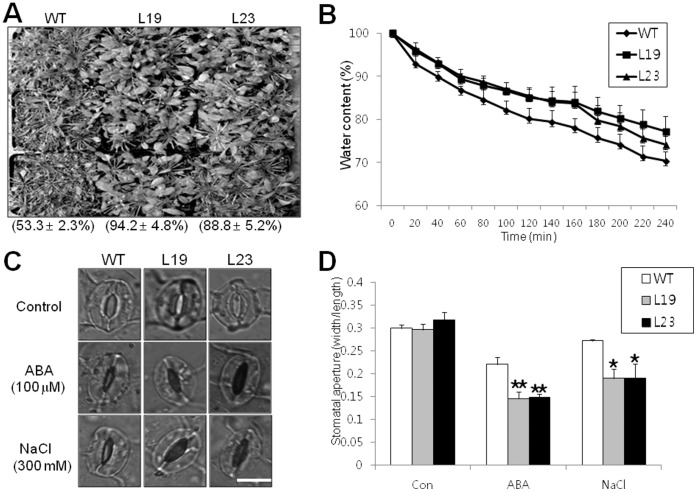
Overexpression of *VrUBC1* enhances drought tolerance and stomatal closure in response to ABA and osmotic stress in Arabidopsis. (A) Four-week-old, soil-grown wild-type and *35S:VrUBC1* Arabidopsis transgenic plants were kept in a growth chamber without watering for 14 d. Photographs were taken 3 d after rewatering. (B) Differential transpiration water loss between the wild-type and the *35S:VrUBC1* Arabidopsis transgenic plants. Detached leaves from 3- to 4-week-old plants grown on soil were incubated at room temperature and fresh weight (FW) was measured at the time intervals indicated. Water content was calculated from percentage of FW compared with weight at zero time. Error bars, mean ± SD of three replicates. (C) Stomatal aperture of the wild-type and *35S:VrUBC1* Arabidopsis transgenic plants. Stomatal guard cells were observed by light microscopy in epidermis from wild-type and *35S:VrUBC1* Arabidopsis transgenic plants treated with NaCl (300 mM) and ABA (100 µM) for 3 h. Bar = 10 µm. (D) Measurement of stomatal aperture of wild-type and *35S:VrUBC1* Arabidopsis transgenic plants. Data are mean ratios of width to length ± SD of three independent experiments (n = 60).

To investigate the possible role of *VrUBC1* in the drought stress response, whole plant survival rates of *35S:VrUBC1* plants under water-deficit conditions were determined (Figure 5). When 4-week-old plants were deprived of water for 14 d, 94.2% (L19) and 88.8% (L23) of the *35S:VrUBC1* plants survived, but only 53.3% of the wild-type plants survived (Figure 5A). This result indicates that the *35S:VrUBC1* transgenic plants were more resistant to drought stress. Additionally, transpiration rates of the transgenic plants were compared with those of the wild-type (Figure 5B). Over 4 h, the fresh weight loss of detached leaves of *35S:VrUBC1* plants was approximately 20% (L19) and 24% (L23), but that of the wild-type was 28% (Figure 5B), suggesting that *VrUBC1* overexpression promoted stomatal closure under water deficit conditions. Stomatal closure is a key ABA-controlled process that determines the rate of transpiration during dehydration stress [46]. To investigate whether *VrUBC1* is involved in ABA-related stomatal closure, we treated leaves of the wild-type and *35S:VrUBC1* plants with ABA (100 µM) or NaCl (300 mM) and analyzed stomatal aperture (Figure 5C and 5D). The guard cells of *35S:VrUBC1* plants showed increased stomatal closure in response to ABA or NaCl compared to the wild-type (Figure 5C and 5D). Thus, this implicates that *VrUBC1* may play a crucial role in drought stress tolerance by ABA-mediated guard cell control.

**Figure 6:**
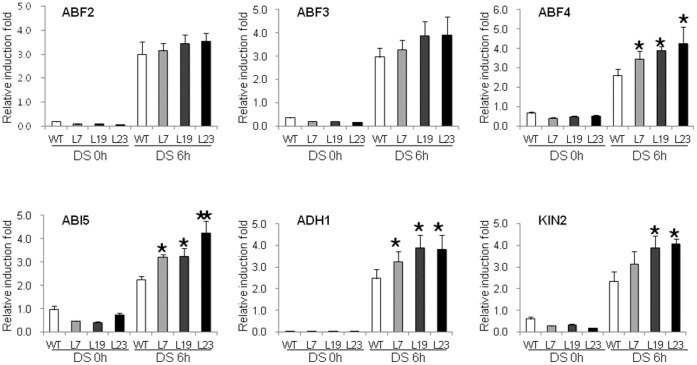
Real-time qRT-PCR analysis of drought-stress maker genes. Total RNA was extracted from the treated tissues and analyzed by real-time qRT-PCR. Light-grown, 4-week-old plants were dehydrated in a vinyl bag for 6 h. Induction patterns of various ABA- and drought-responsive genes (*ABF2*, *ABF3*, *ABF4*, *ABI5*, *ADH1*, and *KIN2*) were analyzed by real-time qRT-PCR. Data represent the fold induction of each gene by dehydration (6 h) relative to the control treatment (0 h). Mean values from three independent technical replicates were normalized to the levels of an internal control, *actin* mRNA. Asterisks indicate the significance of the difference from the values between the wild-type and the *35S:VrUBC1* Arabidopsis transgenic plants as determined by Student’s *t* test (*0.01 ≤ P<0.05, **P<0.01).

The ABA-mediated stress response also triggers stress-responsive gene expression regulated by *ABF*s or *ABREB*s, a small subfamily of ABRE (ABA-responsive element)-binding bZIP (a basic leucine zipper) transcription factors [47]–[50]. Recently, it was shown that *ABI5* belongs to the *ABF* family [51] and acts in postgermination developmental arrest [52]. To investigate the role of *VrUBC1* in transcriptional regulation, the *35S:VrUBC1* transgenic plants and the wild-type were subjected to water deficit stress and then real-time qRT-PCR was used to determine the expression of ABA signaling components, and downstream Arabidopsis ABA-responsive genes (Figure 6). RNA levels of *ABF4*, *ABI5*, *ADH1*, and *KIN2* genes were significantly induced in the *35S:VrUBC1* plants compared to wild-type during dehydration stress (Figure 6). Transcript levels of ABA-responsive and stress marker genes such as *ABF2* and *ABF3* were slightly higher in *35S:VrUBC1* transgenic lines than in the wild-type 12 h after water deficit stress (Figure 6). These results suggested that *VrUBC1* regulates transcription of key ABA-dependent factors such as *ABF4* and *ABI5*, indicating that overexpression of *VrUBC1* thereby contributed to the higher sensitivity in germination to ABA or osmotic stress and increased tolerance to osmotic stress in *35S:VrUBC1* transgenic Arabidopsis.

### VrUBC1 Interacts with the RING E3 Ligase AtVBP1 in Yeast and Plant

**Figure 7:**
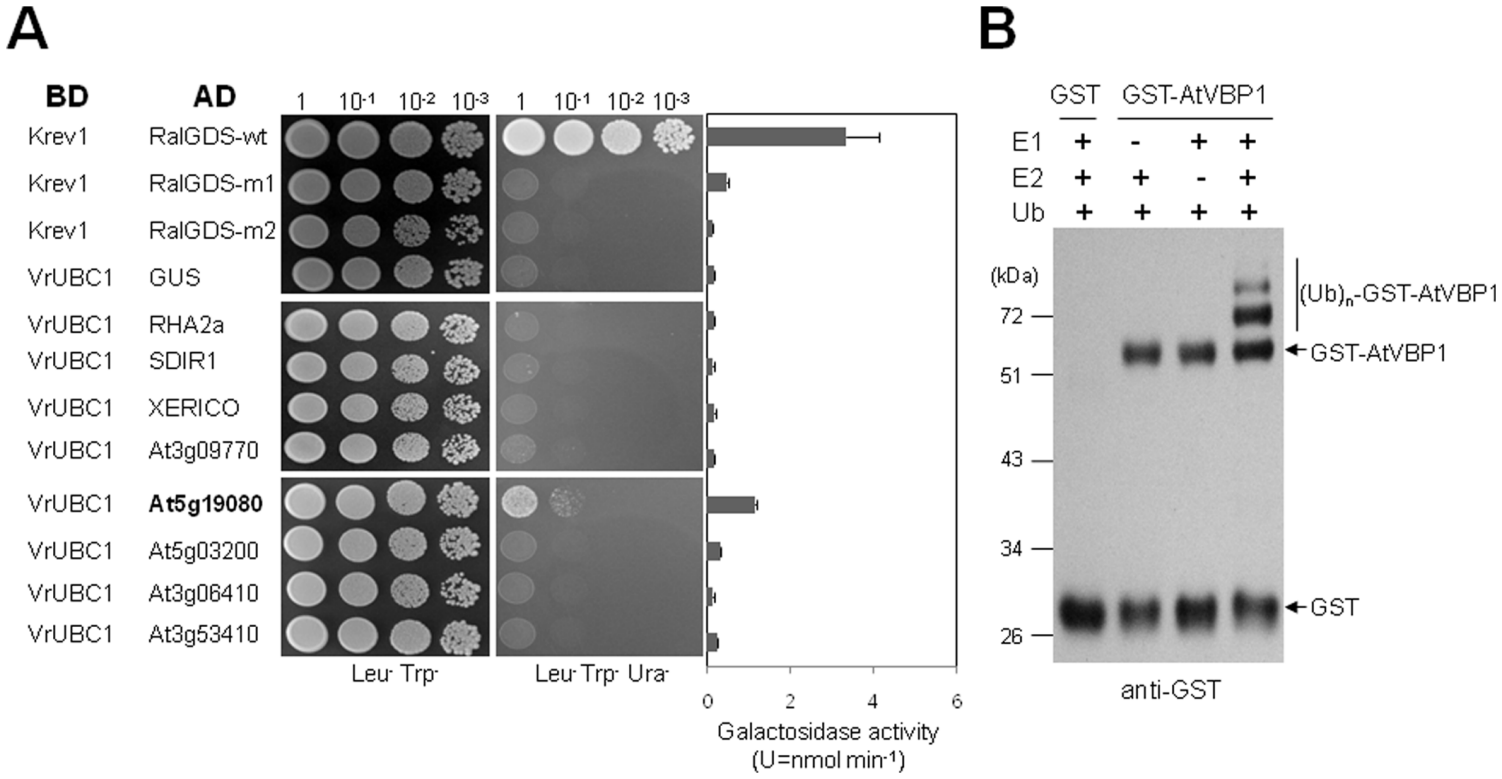
Interaction between E3 ligases and VrUBC1 in yeast and *in vitro* self-ubiquitination of AtVBP1. (A) Protein interactions of VrUBC1 with five C3HC4-type RING E3 ligases and three positive regulators for osmotic tolerance such as RHA2a, SDIR1 and XERICO were analyzed by Y2H system. For the strong positive interaction control, Krev1 (Rap1A, a member of the Ras family of GTP binding proteins) and RalGDS-wt (the Ral guanine nucleotide dissociator stimulator protein) was used. RalGDS-m1 has weak interaction and RalGDS-m2 has no interaction with Krev1. Yeast MaV203 strains containing the indicated plasmid combinations were grown in SD medium without Leu and Trp to an OD600 of 1.0, and 10 µl aliquots of different dilutions (1, 10^−1^, 10^−2^, 10^−3^) were spotted onto selective and non-selective plates (non-selective medium, SD/−Leu/−Trp; selective medium, SD/−Ura/−Leu/−Trp). The combination of plasmids is indicated on the left and dilution series are indicated at the top. β-galactosidase activity was determined in the MaV203 yeast cells cotransformed with the BD/AD plasmids. Data represent means ± SD from three independent experiments. All the experiments were carried out at least in three replications. (B) Purified GST-AtVBP1 was incubated at 30°C for 2 h with VrUBC1 (E2), yeast E1, Ub, and ATP. Samples were separated by SDS-PAGE, and ubiquitinated proteins were detected by immunoblot analysis using anti-GST antibodies.

A number of *in vitro* ubiquitination experiments between Arabidopsis UBC E2s and E3 ligases have been conducted so far, but not *in vivo* [8], [12], [53]. To gain insights into the molecular function of *VrUBC1*, we screened for VrUBC1-interacting proteins from the Y2H (yeast two-hybrid) cDNA library of *A. thaliana*. The E3 ligase that we initially identified (At5g19080) designated as *AtVBP1* (*Arabidopsis thaliana VrUBC1 Binding Partner 1*), showed a moderate interaction with VrUBC1 in the pDEST32/pDEST22 system (Figure 7A). Sequence alignment revealed that there are 4 Arabidopsis RING E3 ligases [53] that are closely related to the RING E3 ligase (At5g19080) identified by our Y2H screen (Figure 7A). These E3 ligases contain the RING domain with the conserved C3HC4 Zn-binding motif and DAR2 (domain associated with RING) [53]. We further used the pDEST32/pDEST22 Y2H system to examine interactions of VrUBC1 with five C3HC4-type RING E3 ligases and a set of other E3 ligases known as positive regulators in osmotic stress tolerance; including *RHA2a*, *XERICO* and *SDIR1* [34], [35], [36] (Figure 7A). VrUBC1 did not show interaction with several positive regulators in osmotic stress tolerance such as RHA2a, XERICO and SDIR1 in yeast (Figure 7A).

Given that VrUBC1, a functional E2, interacts with AtVBP1 in yeast, we examined whether AtVBP1 possesses E3 activity in the presence of VrUBC1 (Figure 7B). Since the GST fusion protein of the full-length AtVBP1 (1–378 aa) was not efficiently expressed in *E. coli*, a truncated version of the GST-AtVBP1 (107–378 aa) recombinant protein was used for the *in vitro* ubiquitination assay (Figure 7B). The reactions were incubated at 30°C for 2 h in the presence of E1, Ub, VrUBC1 (E2) and ATP (Figure 7B). The reaction mixture was terminated and the ubiquitinated proteins were detected by immunoblot analysis using anti-GST antibodies (Figure 7B). As shown in Figure 7B, high-molecular-mass ubiquitinated bands were produced by AtVBP1, indicating that bacterially expressed AtVBP1 is ubiquitinylated by the VrUBC1 (E2) *in vitro*.

### Subcellular Colocalization of VrUBC1 and AtVBP1 in Plant Cells

**Figure 8:**
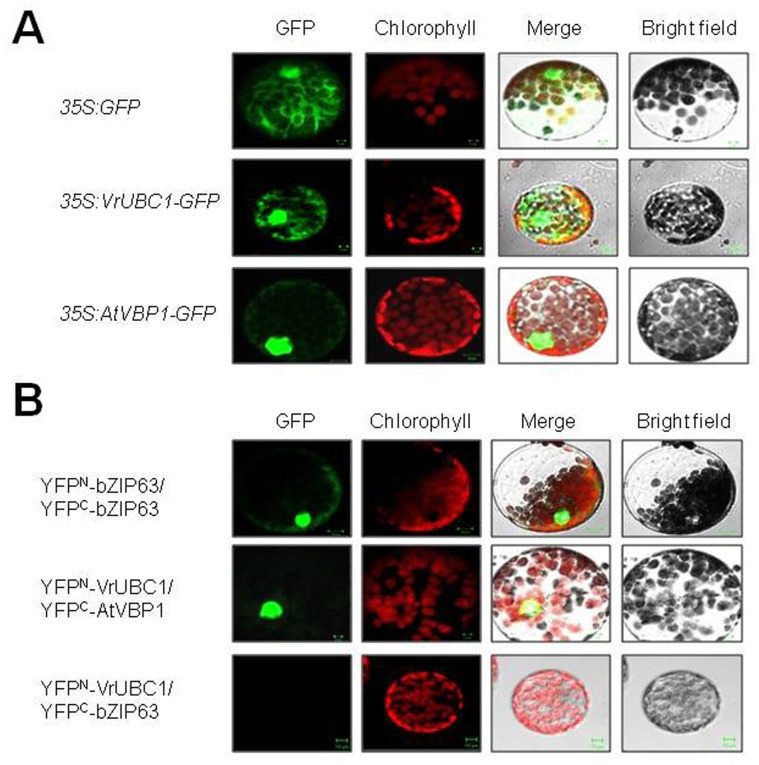
Subcellular localization of GFP-fusion proteins and BiFC visualization of the interaction between VrUBC1 and AtVBP1 in *Agrobacterium*-infiltrated tobacco (*Nicotiana benthamiana*). (A) The *35S:GFP*, *35S:GFP-AtVBP1*, and *35S:AtVrUBC1-GFP* constructs were transformed into tobacco leaves via *Agrobacterium*-infiltration. Protoplasts were isolated from the infiltrated leaves after 36 h. Localization of fusion proteins was visualized by confocal microscopy. (B) YFP^N^ fusions of VrUBC1 and YFP^C^ fusion of AtVBP1 were coexpressed in tobacco leaves as previously described [66]. Epifluorescence from the interaction between YFP^N^ fusions of VrUBC1 and YFP^C^ fusion of AtVBP1 was observed in the nucleus of the protoplasts (scale bar = 20 µm). BiFC of Arabidopsis bZIP63 dimerization is shown as an interaction control as previously described [66]. Coexpression of *YFP^N^-VrUBC1*/*YFP^C^-bZIP63* is shown as a negative control.

To investigate the subcellular localization of VrUBC1 and AtVBP1, we conducted an *in vivo* targeting experiment using fusions of VrUBC1, or AtVBP1 with enhanced green fluorescent protein (GFP) as a fluorescent marker in a transient expression assay (Figure 8A). Each coding region was fused to the N-terminal region of GFP in-frame under the control of the cauliflower mosaic virus (CaMV) 35S promoter. The resulting constructs were introduced into *N. benthamiana* by *Agrobacterium*-mediated transformation [54]. Localization of the fusion protein was then examined by confocal laser scanning microscopy (Figure 8A). Green fluorescence associated with the VrUBC1-GFP fusion protein was localized to the cytosol and the nucleus and the AtVBP1-GFP fusion protein was found exclusively in the nucleus (Figure 8A). PSORT (http://www.psort.org/) predicted that VrUBC1 may be targeted to the cytoplasm and AtVBP1 to the nucleus, in agreement with the subcelluar localization results of the GFP fusion protein observed in Figure 8A.

We confirmed the interactions seen in the Y2H system by directly visualizing the interaction between VrUBC1 and AtVBP1 in living plant cells using BiFC (Figure 8B). To this end, we transiently transformed tobacco (*N. benthamiana*) with *pE-SPYNE*/*pE-SPYCE* constructs (Figure 8B). *YFP^N^-bZIP63*/*YFP^C^-bZIP63* was used as a positive control for the interaction in plants (Figure 8B). Cells transformed with any combination of empty vectors produced no or only background fluorescence (data not shown), but a strong signal in the nucleus was observed when either *35S:YFP^N^-VrUBC1* was coexpressed with *35S:YFP^C^-AtVBP1* (Figure 8B). In the tobacco leaves infiltrated with BiFC constructs, YFP fluorescence appeared exclusively inside the nucleus, in agreement with the observation that AtVBP1-GFP is mainly targeted to the nucleus (Figure 8B). Coexpression of *YFP^N^-VrUBC1*/*YFP^C^-bZIP63* was examined as a negative control (Figure 8B). Fluorescence signal was not observed in the protoplast coexpressing *35S:YFP^N^-VrUBC1*/*35S:YFP^C^-bZIP63* (Figure 8B). After taking data into consideration, it can be concluded that VrUBC1 specifically interact *in vivo* with AtVBP1, which is localized in the nucleus.

### Molecular Characterization of *AtVBP1* in the Osmotic Stress Response

To understand the functional relevance of *AtVBP1* on the osmotic stress response, we used real-time quantitative (q)RT-PCR to examine the expression of *AtVBP1* in response to abiotic stresses and ABA treatment (Figure S3 in File S1). *AtVBP1* transcripts were most significantly up-regulated at 12 h in response to dehydration, high-salinity stresses or ABA application (Figure S3 in File S1). qRT-PCR results demonstrated that *AtVBP1* mRNA showed 7-fold induction by dehydration stress, and 2-fold induction in response to high-salt and ABA treatments (Figure S3 in File S1). The *RAB18* gene was used as a positive control for abiotic stress and ABA, respectively (Figure S3 in File S1). Collectively, the data from Figure S3 in File S1 show that expression of *AtVBP1* is mildly responsive to osmotic stress. These results raise the possibility that *AtVBP1* is involved in osmotic stress tolerance.

**Figure 9:**
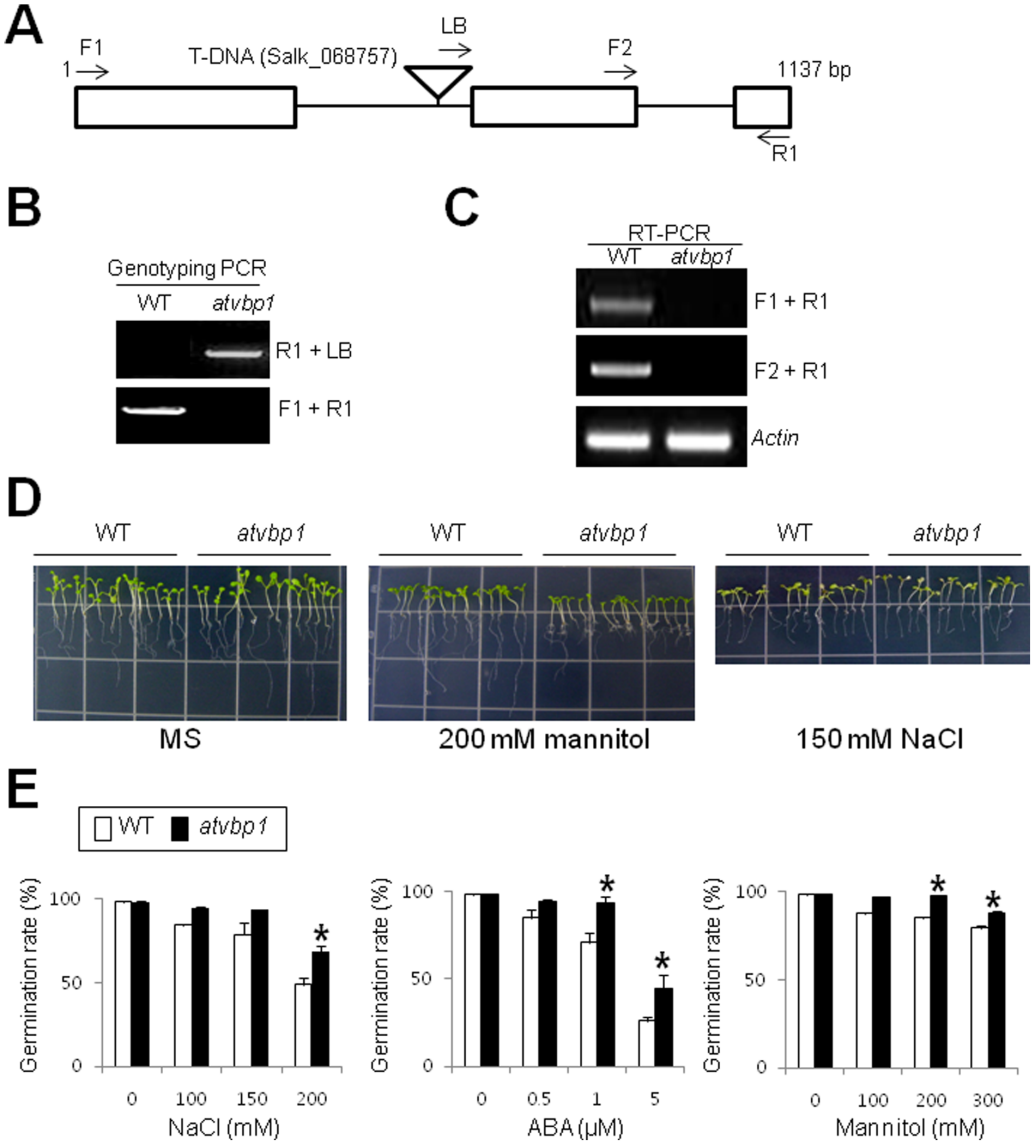
Identification and phenotypic characterization of *atvbp1* mutant plants. (A) Schematic structure of the *atvbp1* allele with the T-DNA insertion. The triangle indicates the T-DNA, white bars indicate coding regions, and the solid line represents introns. Gene-specific (F1, F2, and R1) and T-DNA specific (LB) primers used in genotyping and RT-PCR are indicated with arrows. (B) Genotyping PCR of the *atvbp1* loss-of-function mutant plant. The T-DNA-specific and gene-specific primer sets used for genomic PCR are shown at right of the agarose gel. (C) RT-PCR analysis of *AtVBP1* in the wild-type and *atvbp1* plants. Two primer sets for RT-PCR are indicated at right on the agarose gel. *Actin* transcript levels were used as loading controls. Primers used in genotyping PCR and RT-PCR are listed in Table S1 in File S1. (D) Five-d-old seedlings of the wild-type and *atvbp1* plants were transferred to MS medium containing 2% (w/v) sucrose and 0.8% (w/v) phytoagar supplemented with either mannitol (200 mM) or NaCl (150 mM) and incubated under short day conditions in a growth chamber for 5 days. (E) Seed germination percentage of the indicated lines grown on different concentrations of NaCl, mannitol, or ABA, was recorded at 4 d after the end of stratification. Data show the mean ± SD of three replicates. At least 100 seeds per line were measured in each replicate. Asterisks indicate the significance of the difference from the values between the wild-type and *atvbp1* plants determined by Student’s *t* test (*0.01 ≤ P<0.05, **P<0.01).

To investigate the function of *AtVBP1*, we identified a T-DNA tagged mutant of *AtVBP1*. The *AtVBP1* gene (At5g19080) consists of 1,137 bp with three exons and two introns (Figure 9A). The T-DNA insertion was mapped to the first intron in *AtVBP1* (Figure 9A). Homozygous *atvbp1* mutant plants were verified by genotyping PCR using LB, F1 and R1 primers (Figure 9B). RT-PCR with forward primer F1 or F2 and reverse primer R1 showed that *AtVBP1* mRNA was not detected in the *atvbp1* mutant seedlings (Figure 9C). The *AtVBP1* gene encodes a 378-amino acid protein with a predicted molecular mass of 42.31 kD. AtVBP1 shares a relatively low degree of amino acid sequence identity with other Arabidopsis RING proteins (68% identical to At3g06140, 49% to At3g09770, 47% to At3g53410 and 40% to At5g03200). Growth rates of the wild-type and *atvbp1* seedlings were investigated under osmotic stress conditions (Figure 9D). Five-d-old wild-type and *atvbp1* mutant seedlings were transferred to media containing 150 mM NaCl or 200 mM mannitol and grown for 5 d (Figure 9D). The *atvbp1* mutant displayed greatly reduced growth in the presence of mannitol (200 mM) compared to the wild-type plants, but did not show any difference in growth under NaCl (150 mM) stress (Figure 9D).

To further examine the role of *AtVBP1* in osmotic stress, we examined seed germination rates of wild-type and *atvbp1* mutants in the presence or absence of NaCl, mannitol or ABA (Figure 9E). To further measure germination rates, approximately 100 seeds were plated on full-strength solid MS medium supplemented with different concentrations of NaCl (0, 100, 150, or 200 mM), mannitol (0, 100, 200, or 300 mM) or of ABA (0, 0.5, 1, or 5 µM) (Figure 9E). After 4 d of stratification, germination rates were determined (Figure 9A). The *atvbp1* mutants displayed higher rates of germination than wild type on media supplemented with NaCl, mannitol, or ABA (Figure 9E). With low concentrations of NaCl (100 and 150 mM), mannitol (100 mM) and ABA (0.5 µM), there was no significant difference in germination rate between the wild-type and *atvbp1* mutants (Figure 9E). In the presence of 200 mM NaCl, 48.8% of the wild-type seeds germinated, but 68.1% of *atvbp1* seeds germinated at 4 d (Figure 9E). In case of mannitol, a much higher difference in germination rates was observed between the wild-type (84.8%) and *atvbp1*mutants (97.2%) in the presence of 200 mM mannitol (Figure 9E). A significant difference in germination rates between the wild-type (79%) and *atvbp1* mutants (87.8%) was also observed in the presence of 300 mM mannitol (Figure 9E). The difference in germination rates between wild-type and *atvbp1* was larger with increasing concentrations of ABA (Figure 9E). In the presence of 1 µM ABA, the germination rate of wild-type (70.7%) was much lower than that of *atvbp1* (93.4%) and in the presence of 5 µM ABA, the germination rate of wild-type (26%) was lower than that of *atvbp1* (44%) (Figure 9E). Taken together, these results indicate that *AtVBP1* is involved in ABA signaling during germination and seedling growth under osmotic stress conditions. This study represents the first report linking the expression of a *UBC E2* to its interacting partner in osmotic stress tolerance in plants.

## Discussion

### *VrUBC1* Plays an Important Role in Seed Germination and Osmotic Stress Tolerance

Ubiquitin-dependent protein degradation plays a crucial role in the signal transduction pathways of stress responses and in plant development [55]. UBC E2s (37 in *Arabidopsis*) belong to a multigene family and share high sequence similarities with each other; indeed many may be functionally redundant [12]. Therefore, the single knock-out mutants may show no phenotype [12]. Consequently, it is hard to find functional studies of UBC E2 function in higher plants. *AtUBC2*, a structural homolog of the *RAD6* gene of *S. cerevisiae*, partially complements the UV sensitivity and reduced growth rate of *rad6* mutants at the elevated temperatures [56], [57]. Further studies using the *atubc1-1/atubc2-1* double mutant revealed that the double mutants displayed a dramatically reduced number of rosette leaves, an early-flowering phenotype and reduced transcript levels of a set of floral repressor genes [8]. Several functional analyses have employed the heterologous expression of various UBC E2s in Arabidopsis [21], [22]. Overexpression of soybean *GmUBC2* or peanut *AhUBC2* enhanced drought and salt tolerance by modulating abiotic stress-responsive gene expression in Arabidopsis [21], [22]. Ion antiporter genes such as *AtNHX1* and *AtCLCa*, a proline biosynthetic key enzyme, *AtP5CS*, and copper chaperone for superoxide dismutase gene *AtCCS*, were significantly induced in the *GmUBC2* overexpression transgenic plants [21]. *VrUBC1* (subgroup VI) does not share any sequence similarity to the soybean *GmUBC2* (subgroup III) similar to *S. cerevisiae RAD6*, which is implicated in post-replication repair of UV-damaged DNA, induced mutagenesis, and sporulation [21], [55], [56]. Constitutive expression of *AhUBC2* resulted in upregulation of stress-responsive genes including *P5CS1*, *RD29A* and *KIN1*, but *NCED3*, *ABF3*, *RD29B* and *RD22* genes were not affected in the overexpression plants [22]. *VrUBC1* and *AhUBC2* belong to UBC E2 subgroup VI (Figure S1B in File S1), but the molecular function of *VrUBC1* may be different from that of *AhUBC2* in abiotic stress responses. Compared to *AhUBC2* overexpressing transgenic plants, transcription of ABA-dependent signaling regulators such as *ABF4* and *ABI5* is upregulated in the *35S:VrUBC1* transgenic plants (Figure 6). It is plausible that each UBC E2 has its own interacting E3 ligase partner and therefore each specific E2–E3 interaction results in different phenotypes.

During water deficit, ABA induces stomatal closure, minimizing water loss through transpiration [58]. ABA also inhibits seed germination [46]. *VrUBC1* mRNA levels increased after exposure to salt stress or ABA, suggesting that *VrUBC1* might be involved in plant tolerance to osmotic stress. The *35S:VrUBC1* transgenic plants displayed higher sensitivity to ABA and enhanced osmotic stress tolerance, suggesting that *VrUBC1* may be a signaling component specifically effective during seed germination and drought stress. Expression of several key ABA signaling genes such as *ABF4* and *ABI5*, was significantly upregulated in the *35S:VrUBC1* transgenic plants (Figure 6). Significant upregulation of *ABF4* and *ABI5* may contribute to the increased sensitivity to ABA during germination stages and osmotic stress in the *VrUBC1* transgenic plants (Figure 3–5). bZIP transcription factors such as *ABF4* and *ABI5*, which are involved in ABA-dependent signaling pathways, are implicated in postgermination developmental arrest and abiotic stress tolerance [48], [49], [51]. Overall, we speculate that *VrUBC1* likely acts upstream of ABA-responsive bZIP transcription factors. Together, these data support the idea that *VrUBC1* is an important positive regulator of early events of ABA signal transduction responses during seed germination and in the mature plant.

AtVBP1, a C3HC4-Type RING E3 ligase, interacts with VrUBC1 in ABA signaling in *35S:VrUBC1* Arabidopsis.

The network of human E2–E3 interactions have been extensively analyzed using Y2H assays, thus providing more precise information about the cellular signaling network [59], [60]. A number of *in vitro* ubiquitination experiments between Arabidopsis UBC E2s and E3 ligases have been conducted so far, but not *in vivo* [8], [12], [53]. For example, *XERICO*, encoding a RING-H2 zinc finger E3 ligase, is a positive regulator of ABA biosynthesis for drought tolerance in *Arabidopsis* and was shown to interact with AtUBC8 in yeast [34]. *In vitro* ubiquitination of AtAIRP1, a C3H2C3-type RING E3 ligase, was tested in the presence of several UBC E2s [37]. AtAIRP1 was shown to self-ubiquitinate in the presence of AtUBC8 and AtUBC10, but not in the presence of AtUBC5 and AtUBC13 [37]. In this study, we present a specific E2–E3 interaction *in vitro*, and also *in vivo* (Figure 7 and 8). VrUBC1, a mung bean UBC E2, interacted only with the AtVBP1 protein (E3) out of eight Arabidopsis RING E3 ligases tested in Y2Hassays (Figure 7A). Previously, AtUBC8 was shown to have even broader substrate specificity, interacting with 46 E3 ligases *in vitro* [12], [53]. Furthermore, AtUBC10, 11, 28, and 29, which are highly similar to AtUBC8, showed broad substrate specificity to a large number of RING E3 enzymes, including RING E3s with modified RING domains *in vitro* [12]. Although VrUBC1 shows high sequence identity to AtUBC8 (96%), VrUBC1 did not show the broad interactions with the E3 ligases (Figure 7A). Identification of UBC E2 region that determines the specific interaction with its E3 ligase substrate, as well as the identification of the native mung bean interactors with VrUBC1 remains an interesting topic for future research. For instance, it may be integral to identify the interaction specificties between the partner proteins and Arabidopsis UBCs homologous to VrUBC1 homologs.

To date, there have been reports of several active E3 ligases acting as positive regulators of ABA signaling. However, these genes differ in their functional relationship with ABA molecular responses. *RHA2a* acts in parallel with *ABI5* [35], but *SDIR1* functions upstream of *ABI5* [36]. *AtAIRP1* acts as a positive regulator in ABA-dependent signaling events in response to drought stress [37]. It has been postulated that the E3 ligase, a positive regulator, may ubiquitinate negative regulators of ABA action or signal transduction [37]. However, the interacting target proteins of the E3 ligase have not been identified yet. In contrast to the positive regulators described above, a number of negative regulators (*AIP2 KEG*, *DRIP1*, and *DRIP2*) have been found to interact with target proteins involved in ABA signaling [30], [32]. ABI3 was shown to be a target of AIP2 for ubiquitin-mediated protein degradation [30], [32]. ABI5 protein may be targeted for protein degradation by KEG, which contains a RING-HCa motif, a kinase domain, ankyrin repeats, and HERC2-like repeats [32]. Arabidopsis C3HC4-type RING E3 ligases, *DRIP1*/*DRIP2* act as novel negative regulators in drought-responsive gene expression by inducing ubiquitination of DREB2A to cause its proteasome-mediated degradation [31].

AtVBP1, which interacts with VrUBC1, is a novel C3HC4-type RING E3 ligase, but its specific function has not been identified. We suggest that *AtVBP1* participates in the ABA-dependent drought stress response in Arabidopsis based on the following results. First, *AtVBP1* is induced by osmotic stress and by ABA (Figure S3 in File S1). Second, the loss-of-function *atvbp1* mutant plants were less sensitive to ABA and osmotic stress during germination and postgermination growth (Figure 9D and E). This phenotype (Figure 9D and E) was in sharp contrast to that of the *VrUBC1* overexpression transgenic plants that displayed increased sensitivity to ABA and osmotic stress from germination to maturity (Figure 3 and 4). *In vitro* ubiquitination assays demonstrated that AtVBP1 is ubiquitinylated by the VrUBC1 *in vitro* (Figure 7B). BiFC revealed that the interaction between VrUBC1 and AtVBP1 occurs mostly in the nucleus (Figure 8B). With this in mind, we propose that *AtVBP1* may be involved in protein degradation of a negative regulator in the signaling pathway of osmotic and ABA responses in plants. The decrease in the level of a negative regulator targeted by AtVBP1 might result in increased sensitivity to abiotic stresses. This regulatory interaction between VrUBC1 and AtVBP1 would permit the plant to fine-tune its responses to osmotic stresses and ABA. However, we cannot rule out the possibility that VrUBC1 may interact with other E3 ligase proteins such as AtAIRP1 in plants [37]. More detailed studies about the functional relationship between *AtVBP1* and drought stress adaptation are necessary. Taken together, we hypothesize that VrUBC1 preferentially ubiquitinates AtVBP1 in *35S:VrUBC1* Arabidopsis to mediate the degradation of substrate(s) (yet to be identified) through the ubiquitin-proteasome machinery.

Overall, we propose that mung bean *VrUBC1*, a functional UBC E2, when overexpressed in Arabidopsis, acts as a positive regulator of ABA and osmotic stress by interacting with the AtVBP1 E3 ligase. It is necessary to examine the role of *AtVBP1* in osmotic tolerance to test if transcription of ABA-responsive genes and stomatal closure is altered in *AtVBP1* overexpression transgenic and *atvbp1* mutant plants. Stress-inducible and developmental specific promoters instead of the constitutive promoter may be more applied to maximize the enhancement of stress tolerance in transgenic plants. Furthermore, we are currently trying to identify AtVBP1-interacting proteins by Y2H screening. These experiments will provide a better understanding of the cellular functions of *AtVBP1* with regard to osmotic stress responses in plants.

## Materials and Methods

### Plant Materials, Growth Conditions and Stress Treatments

Mung bean (*V. radiata* L. cv. Nampyoung) and *Nicotiana benthamiana* were grown in a growth chamber at 28°C/24°C with a photoperiod of 16 h at a light intensity of 70 µmol photons m^−2^ s^−1^). Mung bean plants (6-week-old) were treated with low temperature, dehydration, wounding, high salinity stress (100 mM NaCl), and with abscisic acid (ABA) (100 µM) solution as described previously [61]. Arabidopsis (*Arabidopsis thaliana* ecotype Columbia, Col-0) and transgenic plants were grown at 22°C under long-day conditions (16 h light/8 h dark) or short-day conditions (8 h light/16 h dark) with a photosynthetic flux of 130 µmol photons m^−2^ s^−1^. The *atvbp1* T-DNA insertion mutant (SALK_068757) was obtained from the Arabidopsis Biological Resource Center. Genomic DNAs were prepared and gene-specific primers, F1, F2, R1 and T-DNA specific primers LB were used to identify heterozygous and homozygous plants (Table S1 in File S1).

### Sequence Similarity and Phylogenetic Analyses

Protein sequence similarity searches were performed with the BLASTP program (http://www.ncbi.nlm.nih.gov/BLAST/). Selected amino acid sequences were aligned using ClustalX [62]. The phylogenetic tree was generated using the neighbor-joining method [63] in MEGA software version 4. To evaluate the statistical support for tree topology, bootstrap values were calculated using from 100 to 1,000 replicates.

### RNA Gel Blot, RT-PCR and Real-time qRT-PCR Analyses

Isolation of total RNA and RNA gel blot analysis were carried out as described previously [61]. To detect *VrUBC1* gene transcript, 447-bp DNA fragments covering the full-length cDNA of *VrUBC1* was used for probe labeling. For RT-PCR analysis, cDNA was synthesized from 2 µg of total RNA using oligo d(T)_18_ primer, dNTP and M-MLV reverse transcriptase (Invitrogen). PCR amplification was performed using 1 µl cDNA as a template for 26 cycles of: 94°C for 50 s, 55°C for 50 s, and 72°C for 50 s. Real-time qRT-PCR was performed for 40 cycles using 1 µl cDNA as a template and CFX-96™ RealTime system with SYBR Premix (Bio-Rad). qRT-PCR data were analyzed with CFX Manager v2.1 software (Bio-Rad). Data was normalized to *actin* levels. The primers used for PCR analysis are listed in Table S1 in File S1.

### Complementation of *ScUBC4/5* by *VrUBC1*

*S. cerevisiae ubc4/5* mutants were kindly provided by Dr. Stefan Jentsch [41]. For the yeast complementation assay, the ORF of *VrUBC1* (148 aa) was PCR-amplified with CACC-F primer and R primer. PCR products (451 bp) were cloned into the pENTR/D TOPO vector (Invitrogen). *VrUBC1* DNA was then recombined into the gateway destination vector, pYES-DEST52 (Invitrogen). For the control vector, pYES-GFP was used. The yeast *ubc4/5* double mutant was transformed with pYES-GFP, or with pYES-VrUBC1 and then selected on SD media lacking uracil. EGY48 strain (Invitrogen) was used for the wild-type yeast.

### Purification and *in vitro* E2 Activity of VrUBC1 Protein

The ORF of the *VrUBC1* gene was cloned into the Trx and His_6_-tag fusion protein expression vector pBAD202 vector (Invitrogen). The PCR products (448 bp) amplified with CACC-F primer and R-NS primer were directionally inserted into pBAD202. Expression of the Trx-His_6_ fusion protein in *E. coli* strain LMG194 and its affinity purification were performed according to the manufacturer’s instructions.

E2 activities were performed as described by Sullivan and Vierstra [64] (1991). Reaction mixtures containing yeast ubiquitin-activating E1 (100 ng, Sigma), His_6_-tagged ubiquitin (10 µg, Sigma), and the purified recombinant VrUBC1 (0.5 µg) in 20 µl of 50 mM Tris (pH 7.6 at 25°C), 10 mM ATP, and 10 mM MgCl_2_ were incubated at 30°C for 5 min. The reactions were denatured by either boiling for 10 min in the sample buffer with DTT or in the sample buffer including 4 M urea at 30°C for 15 min.

### Transformation Vectors and Construction of Transgenic Plants

To produce the *35S:VrUBC1* transgenic plants, the ORF region of *VrUBC1* (148 aa) was amplified by PCR with CACC-F primer and R primer. PCR products (448 bp) were cloned into the pENTR vector (Invitrogen) and then recombined into the gateway destination binary vector, pH7WG2D (Plant Systems Biology, Belgium; http://www.psb.ugent.be/), in which transgene expression is under the control for the CaMV 35S promoter. Transformation of Arabidopsis was performed by the vacuum infiltration method using *Agrobacterium tumefaciens* strain C58c1 [65]. For the phenotypic analysis, T3 homozygous lines were used. T2 seeds were germinated on MS plates containing 20 µg/ml hygromycin and the resistant plants were transferred to soil to obtain homozygous T3 seeds.

### Germination and Osmotic Stress Tolerance Tests

Each plant was grown in the same conditions, and seeds were collected at the same time. The wild-type and the *35S:VrUBC1* transgenic seedlings (10-d-old) were transferred to MS media with or without NaCl (150 mM) or mannitol (200 mM) and were subsequently grown for 10 d. Root length of the seedlings grown under normal and osmotic stress conditions was measured with five replications. Germination (full emergence of radicles) was scored on MS medium (2% Suc and 0.8% agar) without or with different concentrations of ABA (0, 0.5, 1, 5 µM), NaCl (0, 100, 150 mM) or mannitol (0, 100, 200, 300 mM) as indicated. Plates were chilled at 4°C in the dark for 3 d (stratified) and moved to 22°C with a 16-h-light/8-h-dark cycle. The percentage of seed germination was scored after 4 d with 3 repetitions.

### Drought Stress Treatment, Transpiration Rate and Stomatal Aperture Analysis

For the soil-grown plant drought tolerance test, the 4-week-old plants were subjected to progressive drought by withholding water for 14 d. The test was repeated a minimum of three times. To measure leaf water loss, fully expanded leaves were removed from 4-week-old plants, placed abaxial side up in open Petri dishes at room temperature, and weighed at different time intervals. Leaves of similar developmental stages (third to fifth true rosette leaves) from 4-week-old soil-grown plants were used.

For stomatal closing experiments, the fully expanded leaves from 3- to 4-week-old wild-type and *35S:VrUBC1* plants were excised, and epidermal pieces were peeled from the abaxial surface. The epidermal peels were floated for 2.5 h in stomatal opening solution [36] containing 10 mM KCl, 100 mM CaCl_2_, and 10 mM MES, pH 6.15 and transferred to stomatal opening solution supplemented with ABA (0, 100 µM) or NaCl (0, 300 mM) for 2 h. Epidermal strips were mounted on glass slides and observed with a Zeiss Axiophot microscope. The apertures of stomatal pores were measured using AxioVison4, which calculates the distance between any two points. Over 60 guard cells from each sample were used to measure stomatal aperture.

### Y2H Screen and Specificity Test

The specific interaction between VrUBC1 and RING finger E3 ligases including At5g19080, At3g09770, At5g03200, At3g06410, At3g53410, and other E3 ligases involved in ABA signaling, RHA2a (At1g15100), SDIR1 (At3g55530), and XERICO (At2g04240) was analyzed further. VrUBC1 was cloned into the pDEST32 gateway vector as bait and E3 ligases were cloned to pDEST22 gateway vector as prey (Invitrogen). All the primers used in this experiment are listed in Table S2 in File S1. MaV203 was cotransformed with pBD-VrUBC1 and each pAD-E3 ligase, selected on SD/−Trp/−Leu medium, and allowed to grow for 4 d at 30°C. Transformants were subsequently grown on SD/−Trp/−Leu/−Ura medium or supplemented with X-*β*-Gal for 3 days at 30°C. To measure the specific interaction between bait and prey, *β*-galactosidase assays were carried out using *O*-nitrophenyl-*β*-D-galactopyranoside as a substrate following the manufacturer’s instructions (Invitrogen). MaV203 strain was cotransformed with BD-Krev1/AD-RalGDS-wt for the strong positive control, BD-Krev1/AD-RalGDS-m1 for the moderate positive control, or BD-Krev1/AD-RalGDS-m2, as a negative control.

### Purification and *in vitro* Ubiquitination of AtVBP1 Protein

The N-terminal truncated partial cDNA of *AtVBP1* (108–378 aa) was PCR-amplified with CACC-F2 primer and R1 primer. PCR products were cloned into the pENTR/D TOPO vector. *AtVBP1* DNA was then recombined into the gateway destination vector, pDEST15 (Invitrogen). GST-AtVBP1 fusion proteins were prepared following the manufacturer’s instructions. Ubiquitination assays were carried out as described previously [37]. Reactions (30 µl) containing 50 mM Tris-HCl, pH 7.5; 5 mM MgCl_2_; 0.05 mM ZnCl_2_; 1 mM ATP; 1 mM DTT; 50 ng yeast ubiquitin-activating E1 (Sigma), 2 µg His-tagged ubiquitin (Sigma), 250 ng of the purified recombinant VrUBC1 and 250 ng of GST-AtVBP1 were incubated at 30°C for 90 min. Reactions were stopped by adding 6 µL of 5×SDS-PAGE sample buffer (125 mM Tris-HCl, pH 6.8, 20% glycerol, 4% SDS, and 10% β-mercaptoethanol) and analyzed by SDS-PAGE electrophoresis followed by immunoblotting using monoclonal GST antibodies (Invitrogen).

### Generation of GFP Fusion and BiFC Constructs

The entire coding region of *VrUBC1*, and *AtVBP1* was PCR-amplified with CACC-F primer and R-NS primer (Table S2 in File S1). Each PCR amplified DNA fragment was cloned between *attB1* and *attB2* sites of the pENTR/D TOPO vector, respectively. *VrUBC1*, or *AtVBP1*, was then recombined as an N-terminal fusion of GFP into the gateway destination binary vector, pK7FWG2 (Plant Systems Biology), by a LR recombination reaction.

To carry out BiFC assay, *pE-SPYNE*/*pE-SPYCE* constructs were used [66]. The *YFP^N^-bZIP63* and *YFP^C^-bZIP63* constructs were used as a positive control for the interaction in plants. The ORF region of *VrUBC1*, and *AtVBP1* was PCR-amplified with the CACC-F1 and R1 primers. Each PCR amplified DNA fragment was cloned between *attB1* and *attB2* sites of the pENTR/D TOPO vector, respectively. *VrUBC1* was then recombined as a C-terminal fusion of YFP^N^ into the gateway destination binary vector, *pE-SPYNE*. *AtVBP1* was then recombined as a C-terminal fusion of YFP^C^ into the gateway destination binary vector, *pE-SPYCE*.

### Transient Plant Transformation and Subcellular Localization

Each *35S:GFP*, *35S:VrUBC1-GFP* and *35S:AtVBP1-GFP* construct was transformed into *Agrobacterium* sp. strain C58c1, respectively. For the transient expression of GFP proteins *in planta*, the transformed *Agrobacterium* cells containing *GFP*, *VrUBC1-GFP*, or *AtVBP1-GFP* were inoculated into the leaves of *Nicotiana benthamiana* plants as previously described [54]. Co-infiltration of *Agrobacterium* strains containing the BiFC constructs and the p19 silencing plasmid was carried out as previously described [66]. A Zeiss LSM700 (Germany) confocal microscope was used to observe fluorescence as described previously [60].

## Supporting Information

File S1 Table S1. Primer sequences used for genomic DNA PCR, RT-PCR and Northern blot experiments. Table S2. Primer sequences used for vector constructions. Figure S1. Multiple sequence alignment and phylogenetic analysis of mungbean VrUBC1 and its homologs. (A) Alignment of the deduced amino acid sequences of *VrUBC1* with homologous UBCs. Proteins were aligned using CLUSTALW at the T-coffee website. The active Cys residue is denoted with an inverted delta symbol and the conserved E2 motif [HPN(I/V)(X)3-4GX(I/V/L)C(I/L)X(I/V)(I/L)] is over-lined [45]. Protein sequences are as follows: *A. thaliana* AtUBC10 (DQ027024), *H. sapiens* HsUBCH5D (NP_057067), and *S. cerevisiae* ScUBC5 (P15732). (B) Arabidopsis UBC domain-containing proteins and UBCs orthologous to VrUBC1 from other organisms were retrieved from databases. The protein sequences were used to construct the tree; *P. trichocarpa* (ABK94824), *V. vinifera* (CAO69355), *Malus x domestica* (ACB87920), *P. patens* subsp. *patens* (XP_001764055), *S. tuberosum* (P35135), *S. lycopersicum* (CAA51821), *A. thaliana* AtSCE1a (AEE79711.1), AtRCE1 (AAF19827.1), AtRCE2 (AAD12207.1), AtUBC1 (DQ027016), AtUBC2 (DQ027017), AtUBC3 (DQ027018), AtUBC4 (DQ027019), AtUBC5 (DQ027020), AtUBC6 (DQ027021), AtUBC7 (At5g59300), AtUBC8 (DQ027022), AtUBC9 (DQ027023), AtUBC10 (DQ027024), AtUBC11 (DQ027025), AtUBC12 (DQ027026), AtUBC13 (DQ027027), AtUBC14 (DQ027028), AtUBC15 (DQ027029), AtUBC16 (DQ027030), AtUBC17 (DQ027031), AtUBC18 (DQ027032), AtUBC19 (DQ027033), AtUBC20 (DQ027034), AtUBC21 (DQ027035), AtUBC22 (DQ027036), AtUBC23 (At2g16920), AtUBC24 (DQ027037), AtUBC25 (DQ027038), AtUBC26 (DQ027039), AtUBC27 (DQ027040), AtUBC28 (DQ027041), AtUBC29 (DQ027042), AtUBC30 (DQ027043), AtUBC31 (DQ027044), AtUBC32 (DQ027045), AtUBC33 (DQ027046), AtUBC34 (DQ027047), AtUBC35 (DQ027048), AtUBC36 (DQ027049), AtUBC37 (DQ027050), *B. napus* (ACC38297), *C. annuum* (AAR83891), *P. sativum* (AAA64427), *O. sativa* OsUBC5a (AB074411), OsUBC5b (AB074412), *A. hypogaea* (AAV34697), *A. capillus-veneris* (ABQ65169), *G. max* (AAN03469), *G. thurberi* (AAL99224), *C. reinhardtii* (EDO98738), *P. resinosa* (AAD00911), *C. glabrata* (CAG58813), *S. pombe* (CAA17917), *G. cingulata* (AAC39499), *D. rerio* (NP_001082922), *H. sapiens* HsUBCH5D (NP_057067), *X. laevis* (AAI42570), *S. cerevisiae* ScUBC4 (CAA35528), and ScUBC5 (P15732). Bootstrap values are shown for each node that had >50% support in a bootstrap analysis of 1,000 replicates. Figure S2. Expression analyses and growth phenotypes of the *35S:VrUBC1* transgenic plants. (A) RNA expression of *VrUBC1* was examined by RT-PCR. *Actin* transcript level was used as a loading control. (B) *VrUBC1* RNA expression in the wild-type and the *35S:VrUBC1* transgenic lines analyzed by qRT-PCR. Transcript levels of *VrUBC1* were quantified by qRT-PCR against *actin* transcript level. Each value is the mean ± SD of three independent biological determinations. (C) Three-week-old seedlings of the wild-type and *35S:VrUBC1* Arabidopsis transgenic lines (L7, L9, L19 and L23) were grown in MS medium containing 2% (w/v) sucrose and 0.8% (w/v) phytoagar. (D) Root length was monitored after 3 weeks. The values are the means ± SD (n = 3). This experiment was carried out three times with consistent results. Figure S3. RNA expression of *AtVBP1* and *RAB18* in response to osmotic stress or ABA. Total RNA was extracted from the leaves of Arabidopsis treated with dehydration, NaCl (100 mM) or ABA (100 µM) for the indicated time period (0, 3, 6, 12, 24 h). Induction patterns of *AtVBP1* were investigated by real-time qRT-PCR. *RAB18* was used as a positive control for abiotic stress and ABA. Gene expression was normalized to *Actin* transcript levels as an internal control. Data represent means ± SD from three independent experiments.
(DOCX)
