# Supplementary material for: Overexpression of VrUBC1, a Mung Bean E2 Ubiquitin-Conjugating Enzyme, Enhances Osmotic Stress Tolerance in Arabidopsis
Source: PLoS One. 2013 Jun 18;8(6):e66056. doi: 10.1371/journal.pone.0066056 (PMC3688854; doi:10.1371/journal.pone.0066056)
Supplement: File S1 — Table S1. Primer sequences used for genomic DNA PCR, RT-PCR and Northern blot experiments. Table S2. Primer sequences used for vector constructions. Figure S1. Multiple sequence alignment and phylogenetic analysis of mungbean VrUBC1 and its homologs. (A) Alignment of the deduced amino acid sequences of VrUBC1 with homologous UBCs. Proteins were aligned using CLUSTALW at the T-coffee website. The active Cys residue is denoted with an inverted delta symbol and the conserved E2 motif [HPN(I/V)(X)3-4GX(I/V/L)C(I/L)X(I/V)(I/L)] is over-lined [45]. Protein sequences are as follows: A. thaliana AtUBC10 (DQ027024), H. sapiens HsUBCH5D (NP_057067), and S. cerevisiae ScUBC5 (P15732). (B) Arabidopsis UBC domain-containing proteins and UBCs orthologous to VrUBC1 from other organisms were retrieved from databases. The protein sequences were used to construct the tree; P. trichocarpa (ABK94824), V. vinifera (CAO69355), Malus x domestica (ACB87920), P. patens subsp. patens (XP_001764055), S. tuberosum (P35135), S. lycopersicum (CAA51821), A. thaliana AtSCE1a (AEE79711.1), AtRCE1 (AAF19827.1), AtRCE2 (AAD12207.1), AtUBC1 (DQ027016), AtUBC2 (DQ027017), AtUBC3 (DQ027018), AtUBC4 (DQ027019), AtUBC5 (DQ027020), AtUBC6 (DQ027021), AtUBC7 (At5g59300), AtUBC8 (DQ027022), AtUBC9 (DQ027023), AtUBC10 (DQ027024), AtUBC11 (DQ027025), AtUBC12 (DQ027026), AtUBC13 (DQ027027), AtUBC14 (DQ027028), AtUBC15 (DQ027029), AtUBC16 (DQ027030), AtUBC17 (DQ027031), AtUBC18 (DQ027032), AtUBC19 (DQ027033), AtUBC20 (DQ027034), AtUBC21 (DQ027035), AtUBC22 (DQ027036), AtUBC23 (At2g16920), AtUBC24 (DQ027037), AtUBC25 (DQ027038), AtUBC26 (DQ027039), AtUBC27 (DQ027040), AtUBC28 (DQ027041), AtUBC29 (DQ027042), AtUBC30 (DQ027043), AtUBC31 (DQ027044), AtUBC32 (DQ027045), AtUBC33 (DQ027046), AtUBC34 (DQ027047), AtUBC35 (DQ027048), AtUBC36 (DQ027049), AtUBC37 (DQ027050), B. napus (ACC38297), C. annuum (AAR83891), P. sativum (AAA64427), O. sativa OsUBC5a (AB074411), OsUBC5b (AB074412), A. hypogaea (AAV34697), A. capillus [file pone.0066056.s001.docx]

**Supporting Information**

Table S1 Primer sequences used for genomic DNA PCR, RT-PCR and Northern blot experiments.

| Name | Primer F (5’ – 3’) | Primer F (5’ – 3’) |
| --- | --- | --- |
| *ABF2*  *ABF3*  *ABF4*  *ABI5*  *ADH1*  *KIN2*  *RAB18*  *RD29B*  *VrUBC1*  *actin*  *AtVBP1*  *LB* | GCTAGTGGTGTGGTTCCAAGTTC  AGAACCTCAACCGGTGGAGAGTG  AACTGTGTTCAACAGATGGGTCAG  AGATGACACTTGAGGATTTCTTGGT  TCCACGTATCTTCGGCCATG  GCAACAGGCGGGAAAGAGTAT  TGATGTGACACGAAGAGACACGTTCAC  AAGCTACCTCTCTCCGGAGGTGGAAGTG  ATGGCCTCTAAGCGGATTCTGAAG  TGGACTCTGGTGATGGTGTC  F1 (ATGGGGATCTCCTTAAGCAAGC)  F2 (GAAGCTTTCCCACCAATCAA)  TGGTTCACGTAGTGGGCCATCG | CTGAGCTCTTGCAGCAACCTG  GGAGTCAGATCAGGTGACATCTGG  GGTTCCTCCGTAACTAGCTAATCC  TGGTTCGGGTTTGGATTAGG  TAGCACCTTCTGCAGCGCC  CCGGTCTTGTCCTTCACGAA  AAGACGAGCAGCAGTAGCTCGACGAGG TACGTGTAACAGCAAGGACGAGGGAG  CTAGCCCATTGCATACTTCTGAGTCC CCTCCAATCCAAACACTGTA  R1 (CTTCTCCAAAACCGCTTGAG) |

Table S2 Primer sequences used for vector constructions.

| Gene name | Primer F (5’– 3’) | Primer R (5’– 3’) |
| --- | --- | --- |
| VrUBC1 | F (CACCATGGCCTCTAAGCGGATT)  EcoRI-F (GAATTCATGGCCTCTAAGCGGATT) | R (CTAGCCCATTGCATACTTCTGAGTCC)  R-NS (GCCCATTGCATACTTCTGAGTCC)  SalI-R (GTCGACCTAGCCCATTGCATACTT) |
| AtVBP1  At5g19080 | CACC-F1 CACCATGGGGATCTCCTTAAGCAA)  CACC-F2 (CACCATGATGAGGCCGGTTTAT) | R1 (TCAGTGTTGTTCATCACTACTTTCC)  R1-NS (GTGTTGTTCATCACTACTTTCC) |
| At3g09770  At5g03200  At3g06410  At3g53410  RHA2a  SDIR1 XERICO | CACCATGGGAAACATTAGCAGCAGCGG  CACCATGGGGAATCTGATCAGTTT  CACCATGGGAATCTCCTTTAGCAA  CACCATGGGCAATGTCATAAGCGG  CACCATGGGGCTACAAGGTCAGCT  CACCATGGGTCTATCAAGTCTTCC  CACCATGAGCTTTGTTTTCCGGGG | CTACTCTTGTTCAACTGTTTCTCTCATCC  GTTCTTGTTAATCTCCAA  CTAGTGTTGTTCATCACTACTGTT  CTAGTTCCTGTCGTTGTTGTTCAC  TCAGTGGAGAGAGAAACACGAGAT  TCACCAAACATTAGAAGAAAGCTG’  TCAAACCATGTCGGAAGCATCATC |

**Figure S1** Multiple sequence alignment and phylogenetic analysis of mungbean VrUBC1 and its homologs. (A) Alignment of the deduced amino acid sequences of *VrUBC1* with homologous UBCs. Proteins were aligned using CLUSTALW at the T-coffee website. The active Cys residue is denoted with an inverted delta symbol and the conserved E2 motif [HPN(I/V)(X)3-4GX(I/V/L)C(I/L)X(I/V)(I/L)] is over-lined [45]. Protein sequences are as follows: *A. thaliana* AtUBC10 (DQ027024), *H. sapiens* HsUBCH5D (NP_057067), and *S. cerevisiae* ScUBC5 (P15732). (B) Arabidopsis UBC domain-containing proteins and UBCs orthologous to VrUBC1 from other organisms were retrieved from databases. The protein sequences were used to construct the tree; *P. trichocarpa* (ABK94824), *V. vinifera* (CAO69355), *Malus x domestica* (ACB87920), *P. patens* subsp. *patens* (XP_001764055), *S. tuberosum* (P35135), *S. lycopersicum* (CAA51821), *A. thaliana* AtSCE1a (AEE79711.1), AtRCE1 (AAF19827.1), AtRCE2 (AAD12207.1), AtUBC1 (DQ027016), AtUBC2 (DQ027017), AtUBC3 (DQ027018), AtUBC4 (DQ027019), AtUBC5 (DQ027020), AtUBC6 (DQ027021), AtUBC7 (At5g59300), AtUBC8 (DQ027022), AtUBC9 (DQ027023), AtUBC10 (DQ027024), AtUBC11 (DQ027025), AtUBC12 (DQ027026), AtUBC13 (DQ027027), AtUBC14 (DQ027028), AtUBC15 (DQ027029), AtUBC16 (DQ027030), AtUBC17 (DQ027031), AtUBC18 (DQ027032), AtUBC19 (DQ027033), AtUBC20 (DQ027034), AtUBC21 (DQ027035), AtUBC22 (DQ027036), AtUBC23 (At2g16920), AtUBC24 (DQ027037), AtUBC25 (DQ027038), AtUBC26 (DQ027039), AtUBC27 (DQ027040), AtUBC28 (DQ027041), AtUBC29 (DQ027042), AtUBC30 (DQ027043), AtUBC31 (DQ027044), AtUBC32 (DQ027045), AtUBC33 (DQ027046), AtUBC34 (DQ027047), AtUBC35 (DQ027048), AtUBC36 (DQ027049), AtUBC37 (DQ027050), *B. napus* (ACC38297), *C. annuum* (AAR83891), *P. sativum* (AAA64427), *O. sativa* OsUBC5a (AB074411), OsUBC5b (AB074412), *A. hypogaea* (AAV34697), *A. capillus-veneris* (ABQ65169), *G. max* (AAN03469), *G. thurberi* (AAL99224), *C. reinhardtii* (EDO98738), *P. resinosa* (AAD00911), *C. glabrata* (CAG58813), *S. pombe* (CAA17917), *G. cingulata* (AAC39499), *D. rerio* (NP_001082922), *H. sapiens* HsUBCH5D (NP_057067), *X. laevis* (AAI42570), *S. cerevisiae* ScUBC4 (CAA35528), and ScUBC5 (P15732). Bootstrap values are shown for each node that had >50% support in a bootstrap analysis of 1,000 replicates.

**Figure S2** Expression analyses and growth phenotypes of the *35S:VrUBC1* transgenic plants. (A) RNA expression of *VrUBC1* was examined by RT-PCR. *Actin* transcript level was used as a loading control. (B) *VrUBC1* RNA expression in the wild-type and the *35S:VrUBC1* transgenic lines analyzed by qRT-PCR. Transcript levels of *VrUBC1* were quantified by qRT-PCR against *actin* transcript level. Each value is the mean ± SD of three independent biological determinations. (C) Three-week-old seedlings of the wild-type and *35S:VrUBC1* Arabidopsis transgenic lines (L7, L9, L19 and L23) were grown in MS medium containing 2% (w/v) sucrose and 0.8% (w/v) phytoagar. (D) Root length was monitored after 3 weeks. The values are the means ± SD (n = 3). This experiment was carried out three times with consistent results.


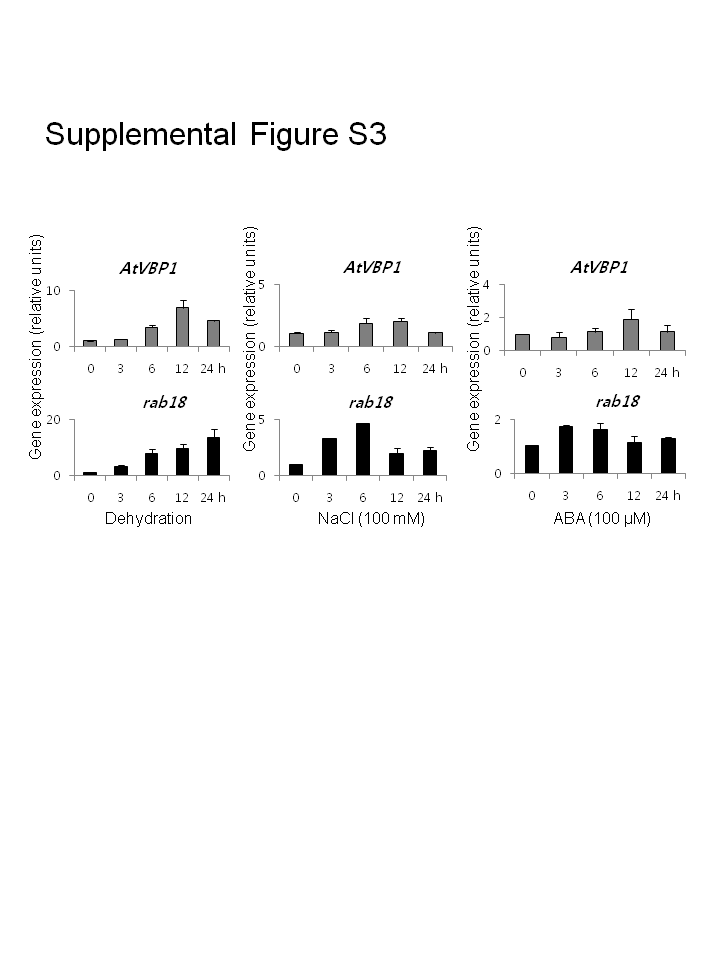


**Figure S3** RNA expression of *AtVBP1* and *RAB18* in response to osmotic stress or ABA. Total RNA was extracted from the leaves of Arabidopsis treated with dehydration, NaCl (100 mM) or ABA (100 µM) for the indicated time period (0, 3, 6, 12, 24 h). Induction patterns of *AtVBP1* were investigated by real-time qRT-PCR. *RAB18* was used as a positive control for abiotic stress and ABA. Gene expression was normalized to *Actin* transcript levels as an internal control. Data represent means ± SD from three independent experiments.
